# Brain‐targeted exosome‐mimetic cell membrane nanovesicles with therapeutic oligonucleotides elicit anti‐tumor effects in glioblastoma animal models

**DOI:** 10.1002/btm2.10426

**Published:** 2022-10-18

**Authors:** Youngki Lee, Minkyung Kim, Junkyu Ha, Minhyung Lee

**Affiliations:** ^1^ Department of Bioengineering College of Engineering, Hanyang University Seoul Korea

**Keywords:** brain‐targeted delivery, glioblastoma, nanovesicle, oligonucleotide, T7 peptide

## Abstract

The brain‐targeted delivery of therapeutic oligonucleotides has been investigated as a new treatment modality for various brain diseases, such as brain tumors. However, delivery efficiency into the brain has been limited due to the blood–brain barrier. In this research, brain‐targeted exosome‐mimetic cell membrane nanovesicles (CMNVs) were designed to enhance the delivery of therapeutic oligonucleotides into the brain. First, CMNVs were produced by extrusion with isolated C6 cell membrane fragments. Then, CMNVs were decorated with cholesterol‐linked T7 peptides as a targeting ligand by hydrophobic interaction, producing T7‐CMNV. T7‐CMNV was in aqueous solution maintained its nanoparticle size for over 21 days. The targeting and delivery effects of T7‐CMNVs were evaluated in an orthotopic glioblastoma animal model. 2′‐*O*‐metyl and cholesterol‐TEG modified anti‐microRNA‐21 oligonucleotides (AMO21c) were loaded into T7‐CMNVs, and biodistribution experiments indicated that T7‐CMNVs delivered AMO21c more efficiently into the brain than CMNVs, scrambled T7‐CMNVs, lipofectamine, and naked AMO21c after systemic administration. In addition, AMO21c down‐regulated miRNA‐21 (miR‐21) levels in glioblastoma tissue most efficiently in the T7‐CMNVs group. This enhanced suppression of miR‐21 resulted in the up‐regulation of PDCD4 and PTEN. Eventually, brain tumor size was reduced in the T7‐CMNVs group more efficiently than in the other control groups. With stability, low toxicity, and targeting efficiency, T7‐CMNVs may be useful to the development of oligonucleotide therapy for brain tumors.

## 1 INTRODUCTION

Glioblastoma represents the majority of primary brain tumors and is one of the leading causes of death worldwide.^1^ Although metastasis from the brain to other organs is rarely reported, the average survival rate of glioblastoma patients is so far despairing.^1^ Currently, the clinical options for glioblastoma include surgical resection or radio/chemotherapy. However, they have failed to significantly improve the 5‐year survival rate. Because of this condition, various research efforts have been made to develop efficient treatments for glioblastoma. Gene therapy has also been investigated as a new modality for the treatment of glioblastoma.^2^ As a therapeutic gene for glioblastoma, the *herpes simplex virus thymidine kinase (HSVtk)* gene has been investigated in preclinical and clinical settings.^2^, ^3^ *HSVtk* gene therapy showed positive effects in animal experiments. In addition, clinical trials evaluating *HSVtk* gene therapy were performed in combination with chemotherapy and radiotherapy. However, the outputs of the trials were not satisfactory.^4^ As another example, small interfering RNAs (siRNA) or antisense oligonucleotides have been investigated for glioblastoma gene therapy. A variety of well‐reported genes that have been targeted for siRNA knockdown are described in the review article,^5^ some of which include *vascular endothelial growth factor (VEGF)*, *hypoxia‐inducible factor‐1α (HIF‐1α)*, and *heat shock protein 70 (HSP70)* genes. These genes are involved in the survival of tumor cells under hypoxic conditions. Even though the efficacies of these siRNAs are well established in animal studies, further studies are required for clinical applications.

Recently, it was suggested that microRNAs (miRs) are involved in tumor cell survival.^6^ A growing accumulation of evidence shows that oncogenic miRs play a crucial role in the progression of cancer, including glioblastoma.^7^ Some miRs were induced in the tumor tissues and promoted the proliferation and survival of tumor cells. In general, miRs bind to 5′‐ or 3′‐untranslated regions (UTRs) of the target mRNAs and then facilitate their degradation. The target genes of tumor‐inducible miRs include pro‐apoptotic and growth arrest genes. As a result of the expression of such miRs, including miR‐21, miR‐221/222, and miR‐128‐1,^8^ apoptosis decreases significantly in tumor tissues. Of those miRs, miR‐21 was investigated intensively. miR‐21 facilitates the mRNA degradation of apoptosis‐related genes, such as *phosphatase and tensin homolog (PTEN)* and *programmed cell death protein 4 (PDCD4)*.^9^, ^10^ In previous studies, anti‐miR‐21 oligonucleotide (AMO21) delivery was shown to decrease miR‐21 levels and consequently the expression of PTEN and PDCD4 in tumor tissue. As a result, proliferation was inhibited, and tumor cell apoptosis was induced.

The effects of AMO21 depend on the delivery efficiency into the glioblastoma. In early‐stage studies, intravenous AMO21 administration shows anti‐tumor effects in animal models.^11^ However, the delivery efficiency of naked AMO21 was not very efficient due to the blood–brain barrier (BBB), suggesting that a high level of administration is required. Due to the BBB, which filters the molecules that enter the brain, it is very challenging to develop an efficient drug delivery platform against brain disease.^12^ To avoid or bypass the BBB, various approaches have been employed. For example, local injection with stereotaxic equipment or intranasal administration has been evaluated for nose‐to‐brain delivery.^13^, ^14^, ^15^ Local injection may be the most efficient because it allows AMO21 to be delivered directly into the tumor site without any significant loss of the injectate. However, direct injection may cause additional damage to normal brain tissue, as well as tumor tissue, because of the injection needle. Noninvasive intranasal delivery of AMO21 may be another option. However, the delivery efficiency by intranasal administration is not consistent and is hard to dose control. In addition, the relatively small volume available for administration and the short retention time are the major disadvantages to nasal delivery.^16^ More recently, brain‐targeted exosomes were developed to deliver AMO21 into the brain. T7‐peptide‐decorated exosomes (T7‐exo) facilitated AMO21 delivery into the brain through the BBB and reduced the tumor sizes.^13^ Exosomes themselves may penetrate into the brain through the BBB, but surface decoration with T7 peptides increased the delivery efficiency of AMO21 into brain tumor sites.

Exosomes are promising extracellular vesicles for the delivery of therapeutic reagents into the brain. However, there are some hurdles to be addressed. First, the production efficiency of exosomes from animal cells is not efficient.^17^ Second, exosomal contents cannot be controlled; the contents of exosomes may vary according to their source cells. Some exosomal contents may induce side effect in the cells. Therefore, careful selection of the exosome source may reduce possible side effects, but the side effects are not yet fully mitigatable. In this study, exosome‐mimetic cell membrane nanovesicles (CMNVs) were developed with isolated cell membranes to address these problems. CMNVs did not contain any encapsulated cell. Also, the isolation of cell membranes produced a large source of CMNVs. In the current study, T7 peptides decorated the surface of CMNVs for brain‐targeted delivery. T7 peptides have been used as a targeting ligand to glioblastoma, since they can target tumor cells by specific binding and also facilitate the transcytosis through BBB.^13^ In this study, T7‐decorated CMNVs (T7‐CMNVs) were evaluated as a systemic delivery carrier of AMO21 into the brain of orthotopic glioblastoma mouse models. The results suggest that T7‐CMNVs may be useful to the delivery of AMO21 in the treatment of glioblastoma.

## 2 MATERIALS AND METHODS

### 2.1 Materials

C6 rat glioblastoma, N2a mouse neuroblastoma, HEK293, and bEND.3 cells were purchased from the Korean Cell Line Bank (Seoul, Korea). Fetal bovine serum (FBS) and Dulbecco's modified Eagle medium (DMEM) were purchased from Welgene (Seoul, Korea). AMO21c sequence was 5′‐UCAACAUCAGUCUGAUAAGCUA‐3′. Both 2′‐*O*‐methyl‐ and cholesterol‐TEG‐modified AMO21 (AMO21c) and Cy5‐labeled AMO21c (Cy5‐AMO21c) were synthesized by Bioneer (Daejeon, Korea). Modification of 2′‐position with the methyl group is a well‐established method to enhance the stability of the antisense oligonucleotides. The modification has advantages such as high nuclease resistance, high affinity to the target mRNAs, and high tissue uptake.^18^ Conjugation of cholesterol to oligonucleotides was also published in the previous reports, in which cholesterol‐conjugated antisense oligonucleotides had inhibitory effects to their counterparts to elicit therapeutic effects.^19^, ^20^, ^21^ Cholesterol‐modified T7 (T7c, cholesterol‐HAIYPRH) and cholesterol‐modified scrambled T7 (scrT7c, cholesterol‐IRHPHYA) were synthesized by Peptron (Daejeon, Korea). The bicinchoninic acid (BCA) assay kit, 3‐[4,5‐dimethylthiazol‐2‐yl]‐2,5‐diphenyltetrazoliumbormide (MTT), and lipofectamine2000 were purchased from Thermo Scientific (Rockford, IL). Extruder kits were purchased from Avanti (Birmingham, AL). Cy5.5 NHS ester, anti‐Ki67 antibody, and the in situ BrdU‐Red DNA fragmentation transferase dUTP nick end labeling (TUNEL) assay kit were purchased from Abcam (Waltham, MA). Anti‐PDCD4 antibody was purchased from Bethyl (Montgomery, TX), and PTEN antibody was purchased from Santa Cruz Biotechnology (Dallas, TX). A miRNeasy FFPE kit was purchased from Qiagen (Valencia, CA). An Iscript cDNA synthesis kit was purchased from Bio‐Rad (Hercules, CA). The SensiFAST SYBR No‐ROX Kit was purchased from Bioline (Boston, MA).

### 2.2 Preparation of cancer‐derived nanovesicles

Cell membranes were isolated as described previously.^22^ Briefly, C6 cells were cultured in 10% FBS containing DMEM until confluency reached nearly 100%. The cells were washed twice with DPBS and harvested with scrapers. After resuspension in DPBS, the cells were treated with a protease inhibitor cocktail and lysed by ultrasonication for 3 min. The inner cellular components were eliminated through sequential centrifugation at 3500*g* for 10 min and 20,000*g* for 25 min at 4°C. The supernatant was centrifuged at 100,000*g* for 50 min at 4°C. The resulting pellet contained cell membranes. The pellet was resuspended in DPBS, ultrasonicated for 3 min, and filtered through a 0.4‐μm polycarbonate filter to prevent contamination and eliminate aggregates. Protein concentration in the cell membranes was measured with the BCA assay. CMNVs and T7‐CMNV were prepared by 10 rounds of sequential extrusion through 1‐μm and 0.2‐μm polycarbonate membranes. Preparation of T7‐CMNVs was performed by adding T7c before extrusion to CMNVs. AMO21c was loaded into CMNVs and T7‐CMNV by simple mixture and incubation for 30 min at room temperature.

### 2.3 Dynamic light scattering and nano tracking analysis

Hydrodynamic size and zeta potential were measured with the Zetasizer Nano ZS system (Malvern Instruments, Malvern, UK). To estimate stability and swelling characteristics, we stored CMNVs and T7‐CMNVs at 4°C and measured the size every 3 days. Nano tracking analysis was also performed to gather complementary information about the characteristics of CMNVs and the actual concentration within the Nanosight system (Malvern Instruments, Malvern, UK).

### 2.4 Loading of AMO21

Various amounts of AMO21c were mixed with a fixed amount of CMNVs and incubated for 30 min at room temperature. AMO21c was loaded onto the CMNVs by hydrophobic interaction between cholesterol and the cell membrane.

To calculate loading efficiency, we labeled AMO21c using Cy5.5 NHS ester according to the manufacturer's manual. Cy5.5‐AMO21c was incubated with either CMNVs or T7‐CMNVs for 30 min at room temperature. The samples were ultracentrifuged at 100,000*g* for 50 min. Then, the pellet was resuspended in DPBS, and fluorescence was measured with a microplate reader (excitation/emission = 673/707 nm). The standard calibration curve was prepared, and loading efficiency was calculated according to the following equation: Loading efficiency (%) = (Amount of AMO21c measured after precipitation)/(Total amount of AMO21c) × 100.

### 2.5 Transmission electron microscopy (TEM)

TEM was performed to investigate the morphology and size of the nanovesicles. CMNV and T7‐CMNV were prepared with or without AMO21c. Mesh copper grids (Ted Pell, Redding, CA) were washed with droplets of distilled water. Then, samples were loaded onto the grids and incubated for 30 min at room temperature. Droplets of samples were soaked with absorbent paper and were dried out in a vacuum chamber. Regarding the negative staining, 2% tungstic acid was used. The samples were observed using TEM (JEM‐2100F, JEOL, Tokyo, Japan).

### 2.6 Flow cytometry

Flow cytometry was performed to evaluate the cellular uptake of nanovesicles in various conditions. C6, N2a, and HEK293 cells were cultured in 10% FBS containing DMEM in a 5% CO_2_ incubator at 37°C. The cells were seeded in a 12‐well microassay plate at a density of 2 × 10^5^ cells per well and incubated for 24 h in a 5% CO_2_ incubator at 37°C. Culture media were replaced afresh prior to transfection. Cy5‐AMO21c‐loaded T7‐CMNVs (T7‐CMNVs/Cy5‐AMO21c) were prepared and added to the cells. The amount of Cy5‐AMO21c was fixed at 0.2 μg/well. After 4 h, the cells were washed twice with DPBS and were harvested in Tris‐EDTA buffer. The cells were centrifuged at 1000*g* for 3 min and resuspended with DPBS in FACS tubes. The proportion of Cy5‐positive cells (%) and mean fluorescence intensity were analyzed by flow cytometry (BD FACS Calibur™, BD Biosciences Immunocytometry Systems, San Jose, CA).

### 2.7 Fluorescence microscopy study

C6 cells were seeded on a chamber slide at a density of 1 × 10^5^ cells per well. T7‐CMNVs/Cy5‐AMO21c were transfected into the cells as described above. The cells were washed twice with DPBS, and the nuclei were stained with DAPI. The stained sections were mounted and observed using AxioScan, a Z1 digital slide scanner (ZEISS, Oberkochen, Germany).

### 2.8 Intracellular trafficking study

Transfection of Cy5‐AMO21c was performed into C6 cells using CMNVs and T7‐CMNVs as described above. To evaluate the mechanism of endocytosis and endosomal escape, various inhibitors were added to the cells at 30 min before transfection (chloroquine = 60 μM, bafilomycin A1 = 200 nM, chlorpromazine = 10 μM, methyl‐*β*‐cyclodextrin = 1.5 mM, amiloride = 200 μM, and filipin III = 0.4 μg/ml). Flow cytometry was performed for endocytosis study as described above. For study of endosomal escape, the cells were treated with lysotracker as manufacturer's manual. Confocal microscopy was performed for visualization of the cells.

### 2.9 Transcytosis study

The in vitro transwell model assay was performed to evaluate T7‐CMNV transcytosis. The bEND.3 mouse endothelial cells were seeded with 10% FBS containing DMEM in a 12‐well transwell plate (Corning, New York, NY) at a density of 4 × 10^5^ cells per well on the apical side of the area to mimic the BBB in vitro. When TEER values reached above 200 Ω cm^2^, T7‐CMNVs/Cy5.5‐AMO21c were added after media replacement. After 6 and 24 h of incubation, bEND.3 cells and media on the basolateral side were harvested. Mean fluorescence intensity was measured either from bEND.3 cells by FACS or from the media using a microplate reader.

### 2.10 MTT assay

C6 cells were seeded in a 96‐well microassay plate at a density of 1 × 10^4^ cells per well, as described above. CMNVs and T7‐CMNVs were added to the cells after medium replacement, ranging from 5 μg/well to 39 ng/well by 2‐fold serial dilution. Lipofectamine was used as a control. After 4 h of incubation, the culture medium was replaced, and the cells were incubated for an additional 20 h at 37°C. Then, 10 μg of MTT per well was added to the cells, which were incubated for an additional 4 h at 37°C. The culture medium was removed, and 100 μl of DMSO was added to the cells. The absorbance at 570 nm was measured using a microplate reader. Cell viability was calculated as follows: Cell viability (%) = (OD_570(sample)_)/(average of OD_570(control)_) × 100.

### 2.11 Hemo‐compatibility assay

Blood samples from Sprague Dawley (SD) rats were harvested and centrifuged at 500*g* for 3 min in EDTA‐containing tubes. Plasma was removed, and red blood cells (RBCs) were washed three times with DPBS. Resuspended RBCs were transferred to microtubes and incubated with 1 μg of naked AMO21c, lipofectamine/AMO21c, CMNV/AMO21c, T7‐CMNV/AMO21c, or scrT7‐CMNV/AMO21c. The control group was treated with DPBS as a negative control. After 1 min of incubation, the samples were diluted with an adequate amount of DPBS and mounted on a glass slide for observation.

### 2.12 Rat glioblastoma model

All experimental animal protocols were approved by the Institutional Animal Care and Use Committee (IACUC) at Hanyang University (accreditation number: 2019‐0205). C6 glioblastoma cells (1 × 10^5^ cells in 10 μl of PBS per animal) were injected into seven‐week‐old male SD rats with stereotaxic equipment under anesthesia. The coordinates of the injection were 3.0 mm lateral from the bregma and 4.0 mm deep from the surface of the skull. The injections were performed at a rate of 0.9 μl/min. After a week, T7‐CMNVs/AMO21c was injected intravenously via the tail vein at a dose of 60 μg AMO21c in 900 μl DPBS. Rats were sacrificed a week after the intravenous injection. Rats were flushed with saline for elimination of blood. After euthanasia, the left ventricle of the rat heart was connected with 0.8 mm inner diameter silicone tube. Using peristaltic pump, 0.9% saline was injected into the rat at 9 ml/min for 5 min. Then, the brains were harvested and the tissues were fixed with 4% paraformaldehyde and embedded with paraffin.

### 2.13 Nissl staining

The paraffin‐embedded brain tissues were cut into 5‐μm‐thick sections. The sections were stained with 0.1% cresyl violet, de‐stained with 70% ethanol and 10% acetic acid, and dehydrated with 100% ethanol and xylene. The images of the sections were analyzed using the Image J software.

### 2.14 Reverse transcription‐polymerase chain reaction (RT‐PCR) for miRNA‐21

Total RNA was extracted from the paraffin‐embedded brain sections using the miRNAeasy FFPE kit. The cDNAs were synthesized with the SensiFAST SYBR‐Lo‐ROX kit (Meridian Bioscience, Memphis, TN). The samples were analyzed using an Applied Biosystems 7500 RT‐PCR System (Applied Biosystems, Waltham, MA). The sequences of the miR‐21 primers used are as follows: forward primer, 5′‐GCCCGCTAGCTTATCAGACTGATG‐3′ and reverse primer, 5′‐GTGCAGGGTCCGAGGT‐ 3′. The sequences of the GAPDH primers used are as follows: forward primer, 5′‐AGACAGCCGCATCTTCT TGT‐3′ and reverse primer, 5′‐CTTGCCGTGGGTAGAGTCAT‐3′.

### 2.15 Immunohistochemistry

The paraffin‐embedded brain tissues were cut into 5‐μm‐thick sections. The sections were deparaffinized and rehydrated sequentially. The samples were washed twice with 0.05% PBST for 5 min and blocked with PBS containing 10% goat serum and 1% BSA for 2 h at room temperature. A rabbit anti‐PDCD4 antibody, a mouse anti‐PTEN antibody, or a mouse anti‐Ki67 antibody was diluted 1:500 in PBS containing 1% BSA. The sections were incubated with the antibodies overnight at 4°C. The samples were washed twice and visualized with AxioScan. TUNEL assay was performed with the Deadend TUNEL system kit according to the manufacturer's manual for the detection of cell death.

### 2.16 Biodistribution

T7‐CMNVs/Cy5.5‐AMO21c were injected intravenously into the rats via the tail vein. The distribution of Cy5.5‐AMO21c was investigated using a Fluorescence in vivo Imaging System (FOBI system, Neo Science, Suwon, Korea). The rat glioblastoma models were prepared as described above, and T7‐CMNV/Cy5.5‐AMO21c was injected at a dose of 60 μg of AMO21c per rat. The rats were sacrificed after 2 or 18 h from injection, and ex vivo fluorescence images were analyzed using the NEOimage software (Neoscience, Suwon, Korea). The brain tissues were cut into 5‐μm‐thick sections. The Cy5.5 signals were observed by fluorescence microscopy. The nuclei were counter‐stained with DAPI.

### 2.17 Blood analysis

Blood samples were harvested from rats at the time of sacrifice. The samples were centrifuged at 500*g* for 3 min, and serum was collected. The serum samples were analyzed with an Automated Chemistry Analyzer (Fujifilm NX700, Tokyo, Japan).

### 2.18 Statistical analysis

Data are presented as mean ± standard deviation, and differences among groups were determined by ANOVA followed by a Tukey's multiple comparisons test. *p*‐values lower than 0.05 were considered statistically significant.

## 3 RESULTS

### 3.1 Preparation and characterization of CMNVs and T7‐CMNVs

**FIGURE 1:**
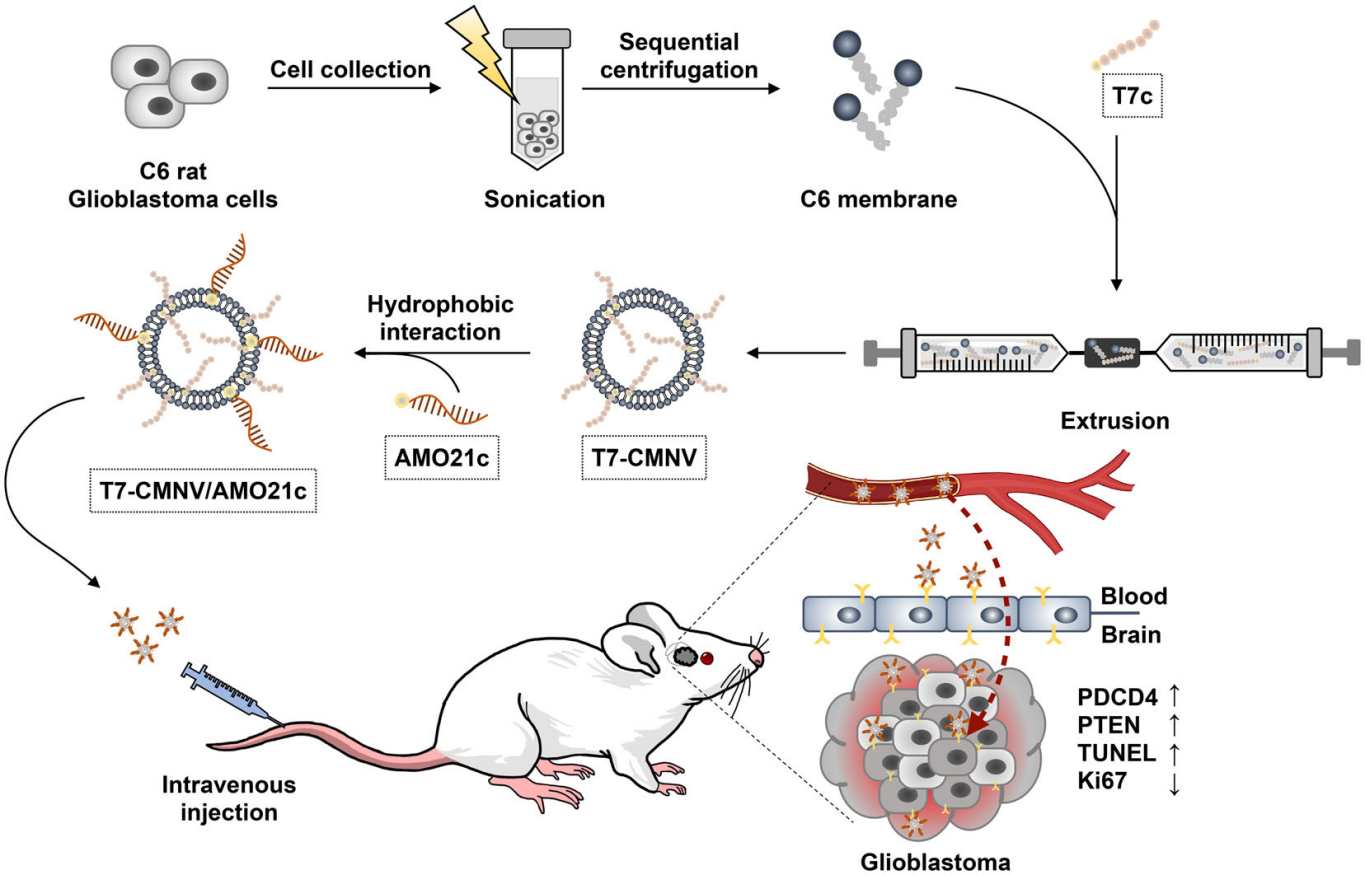
Schematic illustration describing AMO21c delivery with T7‐CMNVs in orthotopic rat glioblastoma models through IV injection.

T7‐CMNVs were prepared with C6 cell membranes to confer AMO21 delivery into the brain. The T7 peptide is a well‐known ligand that binds to transferrin receptors.^23^ Transferrin receptors are over‐expressed in both glioblastoma cells and endothelial cells of the BBB. Therefore, the ligands for transferrin receptors have been extensively studied for glioblastoma targeting and transcytosis into the brain through the BBB. Thereby, BBB transcytosis and cancer targeting could be achieved simultaneously with a single targeting ligand.^24^ The preparation scheme is represented in Figure 1. The C6 cell membranes were isolated by differential centrifugation. Then, the cell membranes were mixed with or without T7c, and the mixture was subjected to extrusion to produce CMNVs or T7‐CMNVs. However, there was a problem of aggregation in the production of T7‐CMNVs. T7c has two moieties that can interact with CMNVs. Cholesterol can interact with CMNVs by hydrophobic interaction, and T7 can interact with the transferrin receptor of CMNVs. Therefore, T7c may contribute to inter‐CMNV interaction as a linker between two CMNVs. The accumulation of these interactions may induce aggregation. Indeed, the preparation of T7‐CMNVs without extrusion induced aggregation in our study. Thus, the extrusion was required to limit the inter‐CMNV interactions of T7c and produce nano‐sized membrane vesicles.

**FIGURE 2:**
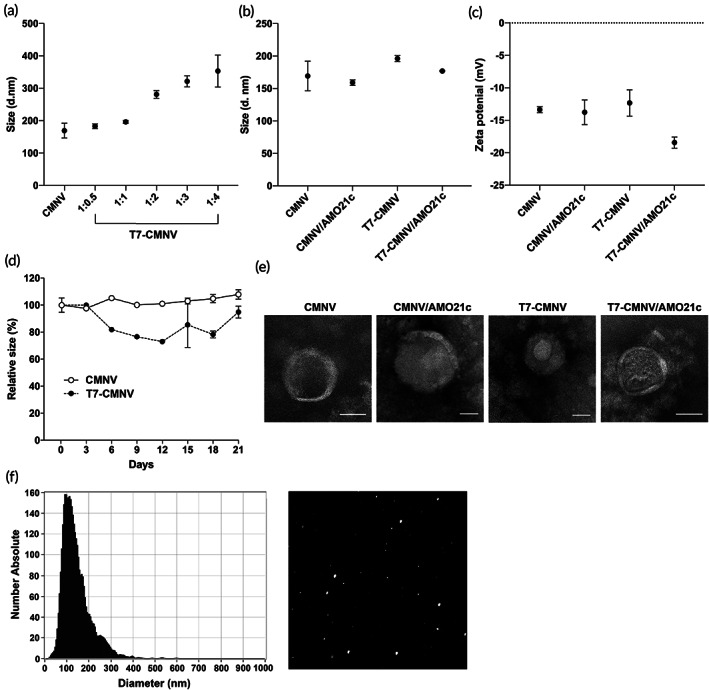
Physical characterization of CMNVs and T7‐CMNVs. (a) Particle sizes of CMNVs and T7‐CMNVs. T7‐CMNVs were prepared with various ratios of T7c. The sizes of T7‐CMNVs were measured by dynamic light scattering. The data are expressed as the mean ± standard deviation of triplicate experiments. (b) Particle sizes of CMNV/AMO21c and T7‐CMNV/AMO21c. CMNVs and T7‐CMNVs were prepared as described in Section 2. The ratio between T7c and CMNVs was 1:1 for T7‐CMNVs. AMO21c was loaded onto CMNVs or T7‐CMNVs at a weight ratio of 1:5 (CMNVs/AMO21c). The sizes of T7‐CMNVs were measured by dynamic light scattering. The data are expressed as the mean ± standard deviation of triplicate experiments. (c) Zeta potential of nanovesicles. The zeta‐potentials of CMNVs, T7‐CMNVs, CMNV/AMO21c, and T7‐CMNV/AMO21c were measured using a zeta potentiometer. The data are expressed as the mean ± standard deviation of triplicate experiments. (d) Stability of CMNVs and T7‐CMNVs in aqueous solution. CMNVs and T7‐CMNVs were incubated at 4°C for up to 21 days. The particle size was measured by dynamic light scattering at the designated times. The data are expressed as the mean ± standard deviation of triplicate experiments. (e) TEM images. Scale bars indicate 100 nm. (f) Nanotracking analysis. CMNVs were analyzed in a nanotracking system. Left panel: size distribution of CMNVs, Right panel: video frame.

The particle sizes of T7‐CMNVs tended to increase along with the ratio of CMNVs and T7c (Figure 2a). T7 peptides on the surface of the CMNVs may increase the particle size. In addition, T7c may contribute to inter‐CMNV interactions, which can increase the size by inducing fusion of two or more CMNVs. However, extrusion might limit the size increase of T7‐CMNVs, and the particle size seemed not to significantly increase at a 1:4 ratio compared with the other tested ratios (Figure 2a).

After producing T7‐CMNVs, AMO21c was loaded onto the vesicles by hydrophobic interaction. The loading of oligonucleotides into the nanovesicles has been performed with electroporation, sonication, or through the usage of transfection reagents. However, the loading efficiency of the oligonucleotides into the nanovesicles was poor, and the loading efficiency by electroporation is around 1%–5%.^13^, ^25^ In this study, AMO21 was conjugated with a cholesterol moiety, producing AMO21c. AMO21c was mixed with CMNVs, so AMO21c was loaded onto the CMNVs by hydrophobic interaction. Although most were loaded onto the surface of the CMNVs, AMO21c was modified at the 2′‐position with a methyl group, so it was not degraded by nucleases in the serum. The loading efficiencies of AMO21c onto CMNVs and T7‐CMNVs were also measured. The loading efficiencies were 14.58 ± 3.08% and 15.1 ± 1.97% for CMNVs and T7‐CMNVs, respectively. The ratio between CMNVs and AMO21c was fixed at 1:5 based on the cellular uptake study (Figure S1).

The particle sizes of AMO21‐loaded CMNVs (CMNV/AMO21c and T7‐CMNV/AMO21c) were measured by dynamic light scattering. The results showed that the particle sizes were not increased following AMO21c loading for both CMNVs and T7‐CMNVs (Figure 2b). The particle size has a significant influence on pharmacodynamics. Since it is difficult for particles with diameters larger than 200 nm to penetrate BBB, the ratio between T7c and CMNVs was fixed at a 1:1 ratio. At this ratio, the average size of T7‐CMNV/AMO21c was 176.96 nm ± 0.65.

The zeta‐potential of the CMNVs was around −13 mV since the cell membranes had negative charges (Figure 2c). The zeta‐potential of the T7‐CMNVs seemed to be slightly higher than that of CMNVs, though the difference between the two groups was not statistically significant. It is speculated that T7‐CMNVs might have a higher zeta‐potential than CMNVs because the charge of T7c is positive due to arginine residues. The loading of AMO21c onto the T7‐CMNVs might have masked the positive charges of T7c, reducing the zeta‐potential (Figure 2c). This effect was not clearly identified in CMNVs, probably due to its lack of positive‐charged peptides, such as T7 (Figure 2c). The negative surface charge of CMNVs may be helpful for circulation since interaction with negatively charged serum proteins, such as albumin, might be reduced due to charge repulsion.^26^

The stabilities of CMNVs and T7‐CMNVs are also important to their therapeutic applications. After CMNVs and T7‐CMNVs were produced, the nanovesicles were stored at 4°C for up to 21 days. Their size stability was evaluated by dynamic light scattering. The results show that the sizes of the CMNVs were not significantly changed during their incubation (Figure 2d). The morphology of the CMNVs was evaluated by TEM, which demonstrated their spherical form (Figure 2e). To reconfirm results from DLS and TEM, we measured the nanovesicles again with NTA (Figure 2f). In addition, by comparing NTA results with those of the typical bicinchoninic acid assay, the concentration of CMNVs was more precisely speculated: approximately 6.15 × 10^11^ ± 1.16 × 10^11^ particles per 1 mg protein.

**FIGURE 3:**
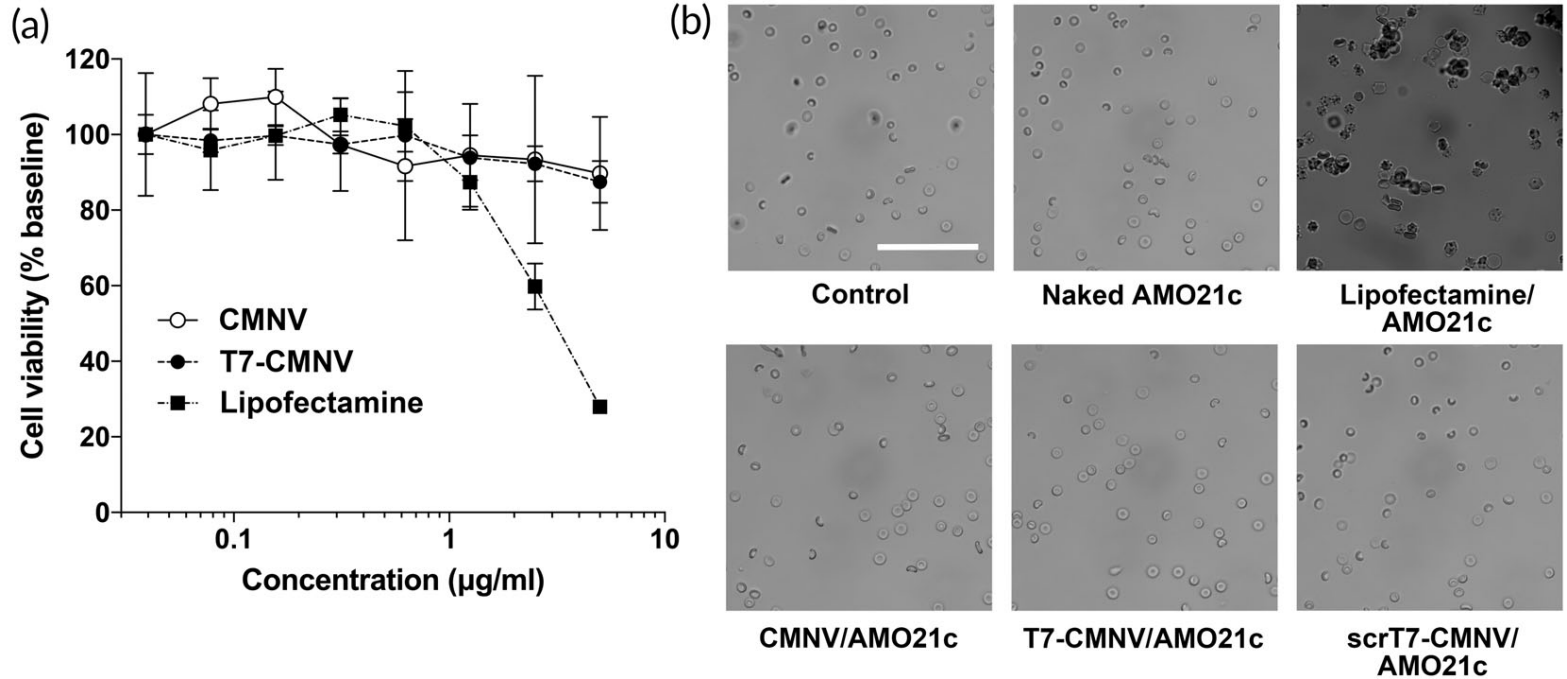
Cytotoxicity assay. (a) MTT assay. C6 cells were incubated with CMNVs, T7‐CMNVs, and lipofectamine at various concentrations for 24 h. Cell viability was measured through the MTT assay. The data are expressed as the mean ± standard deviation of octuplicate experiments. (b) Hemolysis assay. Red blood cells were incubated with naked AMO21c, lipofectamine/AMO21c, CMNV/AMO21c, T7‐CMNV/AMO21c, or scrT7‐CMNV/AMO21c for 1 min. The morphology of the cells was observed using an optical microscope. The scale bar is 50 μm.

The cytotoxicity of CMNVs and T7‐CMNVs was evaluated by MTT and hemolysis assays. As a control for AMO21c carriers, lipofectamine was used. The MTT assay showed that CMNVs and T7‐CMNVs did not induce detectable toxicity in cells up to 5 μg/ml concentration (Figure 3a). However, the toxicity in the lipofectamine‐treated cells increased significantly, starting at 2.5 μg/ml (Figure 3a). The toxicity of cationic lipids may be due to charge interaction with the negatively charged cell membrane. A previous report indicated that the charge interaction of a cationic gene carrier on the surface of a cell membrane induced aggregation and cell membrane rupture, resulting in a decrease in cell viability.^27^ Therefore, the toxicity of lipofectamine may be due to its cationic charge. In the cases of CMNVs and T7‐CMNVs, they had negative surface charges and did not have charge interactions with cell membranes.

The toxicities of CMNVs and T7‐CMNVs were also evaluated through a hemolysis study. CMNV/AMO21c and T7‐CMNV/AMO21c were added to the erythrocytes. The results showed that CMNV/AMO21c and T7‐CMNV/AMO21c did not induce hemolysis (Figure 3b). However, lipofectamine/AMO21c induced hemolysis significantly (Figure 3b). Lipofectamine/AMO21c may have less toxicity than lipofectamine alone because the surface charge of lipofectamine is masked by the negative charge of AMO21c. However, lipofectamine/AMO21c interacted with erythrocyte cell membranes and induced hemolysis.

### 3.2 In vitro AMO delivery of CMNVs and T7‐CMNVs

**FIGURE 4:**
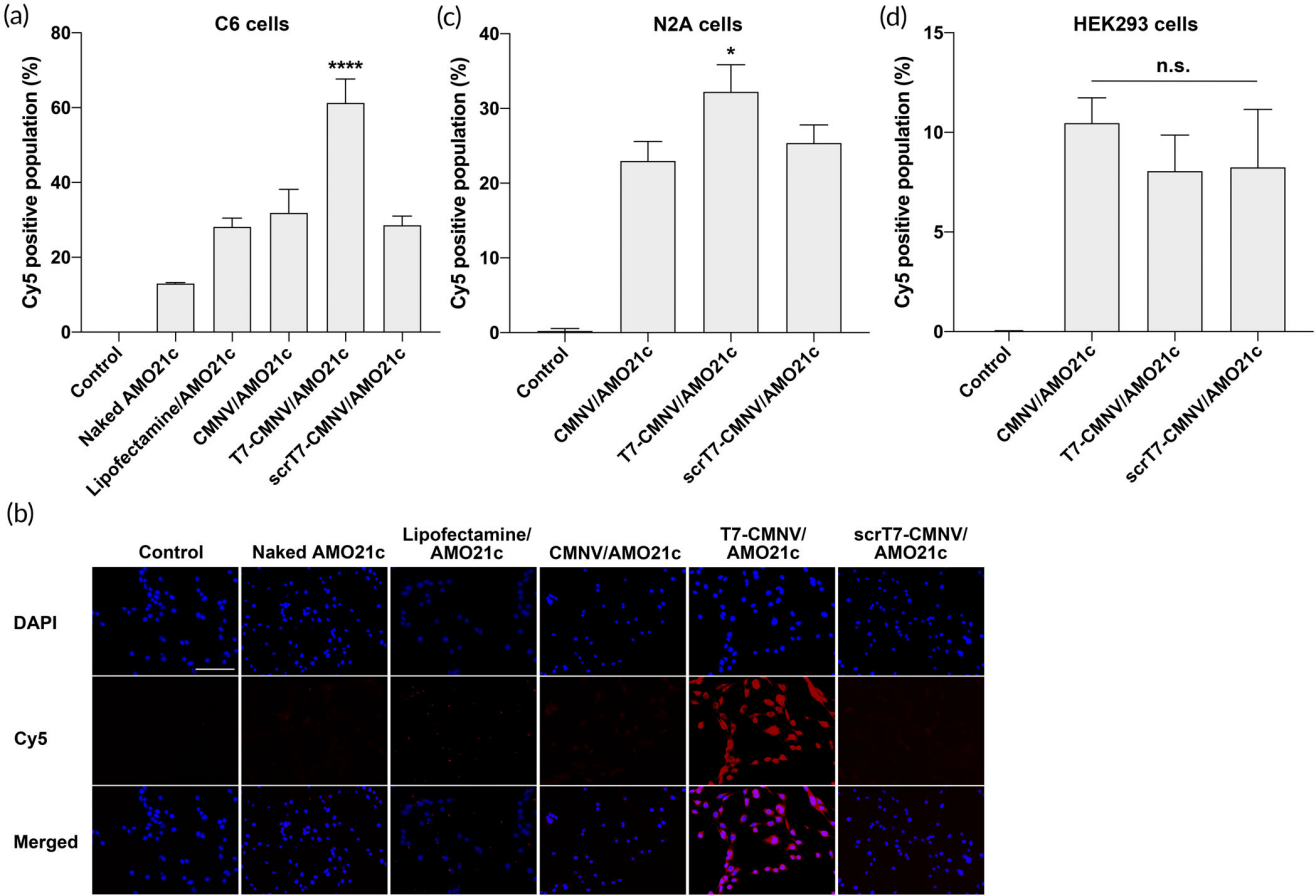
AMO21c delivery efficiency in vitro. (a) Delivery efficiency to C6 cells (flow cytometry). Naked Cy5‐AMO21c, lipofectamine/Cy5‐AMO21c, CMNV/Cy5‐AMO21c, T7‐CMNV/Cy5‐AMO21c, and scrT7‐CMNV/Cy5‐AMO21c were prepared and added to C6 cells. The cellular uptake of Cy5‐AMO21c was measured by flow cytometry. The data are expressed as the mean ± standard deviation of quadruplicate experiments. ****p < 0.0001 as compared to the other groups. (b) Delivery efficiency to C6 cells (fluorescence microscopy). The delivery efficiency of AMO21c to C6 cells was also evaluated by fluorescence microscopy. Nuclei were counter‐stained with DAPI. The scale bar indicates 100 μm. (c) Delivery efficiency to N2A cells. The samples were prepared and added to N2A cells. The cellular uptake of Cy5‐AMO21c was measured by flow cytometry. The data are expressed as the mean ± standard deviation of quadruplicate experiments. *p < 0.05 as compared to the other groups. (d) Delivery efficiency to HEK293 cells. The samples were prepared and added to N2A cells. The cellular uptake of Cy5‐AMO21c was measured by flow cytometry. The data are expressed as the mean ± standard deviation of quadruplicate experiments. n.s., statistically not significant as compared with one another.

The delivery efficiencies of CMNVs and T7‐CMNVs were evaluated with Cy5‐AMO21c. AMO21c‐loaded CMNVs (CMNV/AMO21c) and AMO21c‐loaded T7‐CMNVs (T7‐CMNV/AMO21c) were added to the C6 cells. Naked AMO21c and lipofectamine/AMO21c were used as controls. The cellular uptake efficiency of each sample was measured by flow cytometry. The results showed that T7‐CMNVs had the highest cellular uptake efficiency of AMO21c into C6 glioblastoma cells (Figure 4a). Lipofectamine, CMNVs, and scrambled T7‐CMNVs (scrT7‐CMNVs) had similar uptake efficiencies (Figure 4a). In particular, scrT7‐CMNVs did not increase delivery efficiency compared with CNMVs. This suggests that the increase in T7‐CMNV delivery may be due to the effect of T7 peptides. The results were confirmed by fluorescence microscopy (Figure 4b).

The delivery efficiencies of CMNVs and T7‐CMNVs were compared in different types of cells. In the delivery assay with N2A neuroblastoma cells, T7‐CMNVs had higher delivery efficiencies than CMNVs and scrT7‐CMNVs (Figure 4c). Neuroblastoma was also reported to express transferrin receptors at a higher level than normal cells.^28^ Therefore, T7‐CMNVs may have higher delivery efficiency in neuroblastoma cells due to there being more interactive with transferrin receptors. It was also observed that CMNVs and scrT7‐CMNVs had similar delivery efficiencies to N2A cells. This confirmed that the higher efficiency of T7‐CMNVs was due to the effect of T7 peptides.

As noncancer cells, HEK293 cells were used to evaluate the delivery efficiency of T7‐CMNVs. The same delivery study was performed with Cy5‐AMO21c. The results confirmed that T7‐CMNVs did not induce AMO21c cellular uptake as compared to CMNVs and scrT7‐CMNVs (Figure 4d). HEK293 cells do not express transferrin receptors at a significant level,^28^ and, therefore, the effect of T7 peptides on the delivery efficiency T7‐CMNVs into HEK293 cells was limited. The results indicate that T7‐CMNVs may transfer AMO21c into transferrin receptor‐expressing cells, such as cancer cells and endothelial cells.

**FIGURE 5:**
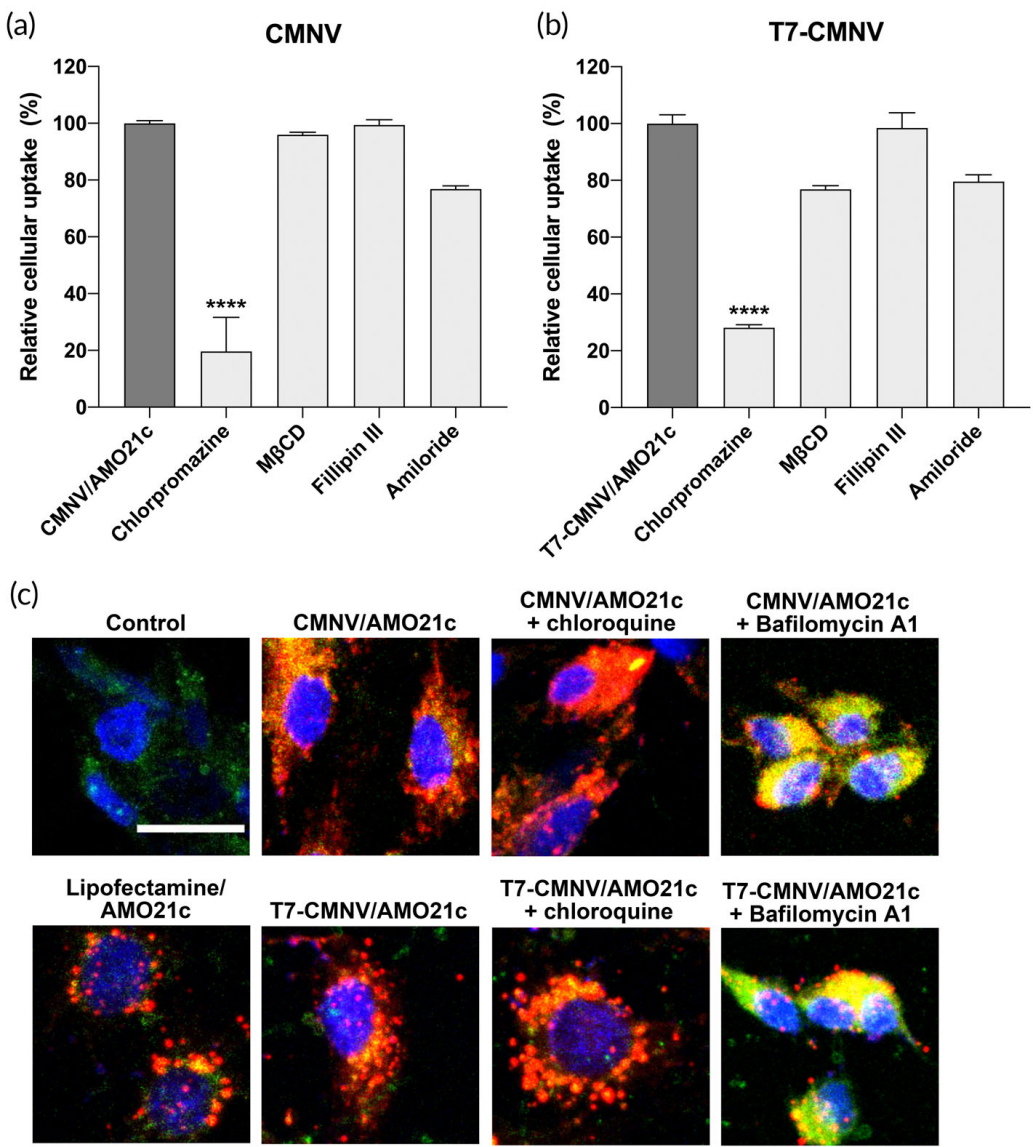
Intracellular trafficking study. The cells were incubated with various inhibitors of intracellular trafficking at 30 min before transfection. CMNV/Cy5‐AMO21c (a) and T7‐CMNV/Cy5‐AMO21c (b) were prepared and added to C6 cells. The cellular uptake of Cy5‐AMO21c was measured by flow cytometry. The data are expressed as the mean ± standard deviation of quadruplicate experiments. ****p < 0.0001 as compared with the other samples. (c) Endosomal escape study. For study of endosomal escape, cells were treated with lysotracker. Confocal microscopy was performed for visualization of the cells.

To investigate the intracellular pathway of CMNVs and T7‐CMNVs, cellular uptake studies were performed after the treatment of inhibitors for intracellular trafficking. The results showed that chlorpromazine reduced the cellular uptake of CMNV and T7‐CMNVs (Figure 5a,b). The results suggest that clathrin‐dependent endocytosis was the major pathway for cellular entry of CMNV and T7‐CMNV. Another important point was the endosomal escape of AMO21c after endocytosis of CMNV/AMO21c and T7‐CMNV/AMO21c. The release from endosomes were investigated with Cy5‐AMO21c. Confocal microscopy studies showed that most of AMO21c was not co‐localized with lysosomes in the lipofectamine/AMO21c, CMNV/AMO21c, and T7‐CMNV/AMO21c groups (Figure 5c). Treatment of the cells with chloroquine induced release of AMO21c slightly in the CMNV/AMO21c and T7‐CMNV/AMO21c groups. In addition, treatment with bafilomycinA1, which was an inhibitor of proton buffering effect, showed that most of AMO21c was colocalized with lysosomes in the CMNV/AMO21c and T7‐CMNV/AMO21c groups. Therefore, it is clear that AMO21c was released from the endosomes after the endocytosis of CMNV/AMO21c, and T7‐CMNV/AMO21c.

**FIGURE 6:**
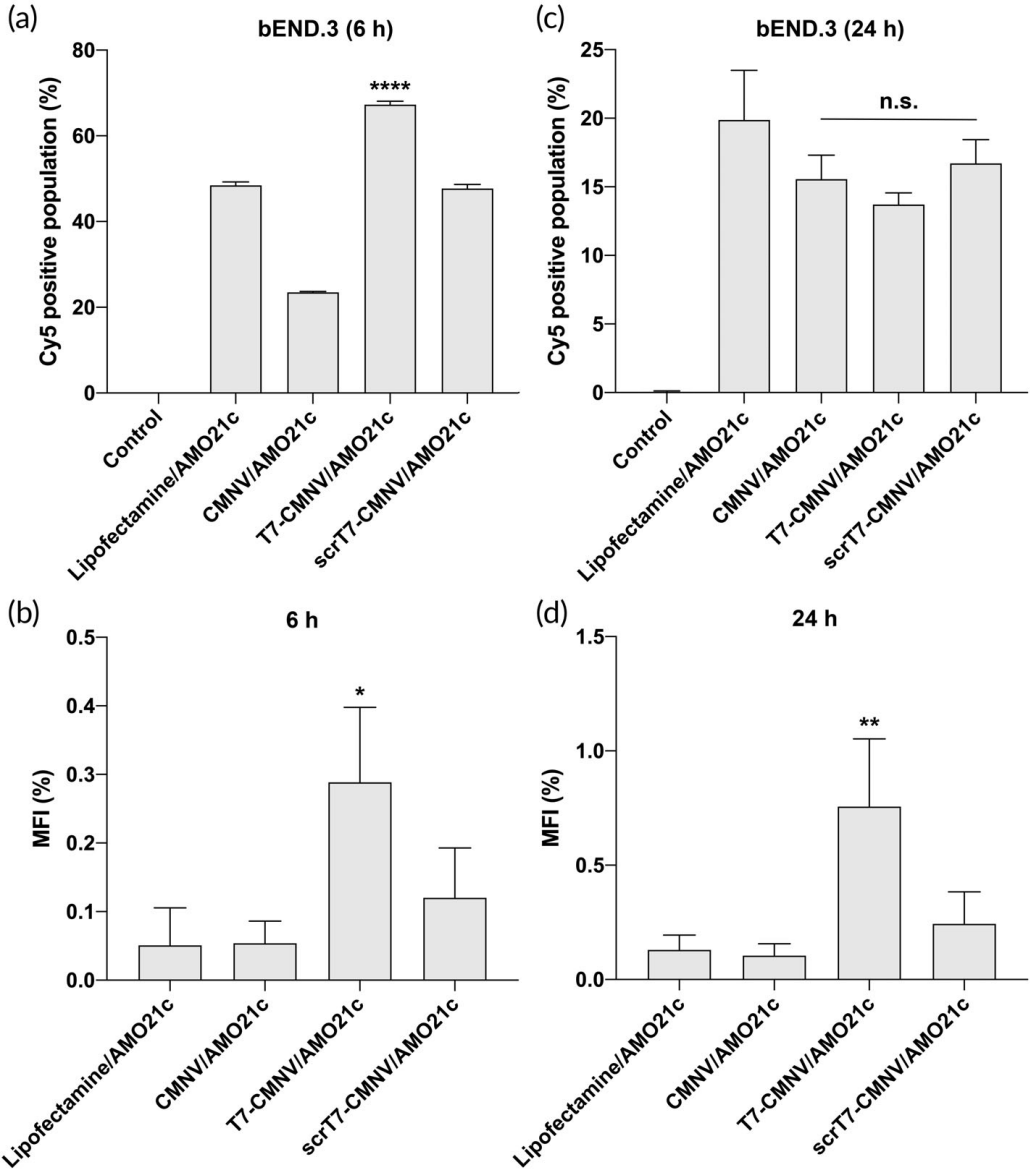
Transcytosis assay. Cy5‐AMO21c uptake was measured in bEND.3 brain endothelial cells. Lipofectamine/Cy5‐AMO21c, CMNV/Cy5‐AMO21c, T7‐CMNV/Cy5‐AMO21c, and scrT7‐CMNV/Cy5‐AMO21c were prepared and added to bEND.3 cells in transwells. Cellular uptake of bEND.3 cells after (a) 6 h and (c) 24 h of incubation was measured with flow cytometry. The mean fluorescence intensity of basolateral media after (b) 6 h and (d) 24 h of incubation was measured with a microplate reader. The data are expressed as the mean ± standard deviation of quadruplicate experiments. ****p < 0.0001, **p < 0.01, and *p < 0.05 as compared to the other groups. n.s., statistically not significant as compared with one another.

To verify transferrin receptor binding and transcytosis ability of T7‐CMNVs, we performed transwell experiments with CMNVs and T7‐CMNVs. bEND.3 mouse endothelial cells were seeded on the apical side of the transwell and cultured for production of endothelial layer. Cy5‐AMO21c‐loaded CMNVs or T7‐CMNVs were added to the bEND.3 cells. Lipofectamine/Cy5‐AMO21c was used as a control. At the designated time, positive fluorescence signals in the bEND.3 cells on the transwells and media from the basolateral side of microassay plates were measured by flow cytometry and a microplate reader. After 6 h of incubation, fluorescence signals in the bEND.3 cells were increased following the addition of T7‐CMNV/AMO21c, compared with CMNV/AMO21c and scrT7‐CMNV/AMO21c (Figure 6a). This result suggests that T7 peptides on T7‐CMNVs increased intracellular delivery of AMO21c into the bEND.3 cells. On the bottom of the microassay wells, media had the highest fluorescence signals in the T7‐CMNV/AMO21c group, suggesting that AMO21cs were delivered into media of the bottom wells by transcytosis of bEND.3 cell layer (Figure 6b). After 24 h of incubation, the fluorescence level of T7‐CMNV/AMO21c‐treated bEND.3 cells was similar to that of CMNV/AMO21c and scrT7‐CMNV/AMO21c (Figure 6c). In addition, the fluorescence level in the media of the T7‐CMNV/AMO21c group was increased further (Figure 6d). The results suggest that the cellular uptake and transcytosis process were facilitated by T7‐CMNVs.

### 3.3 Biodistribution of T7‐CMNV after intravenous administration

**FIGURE 7:**
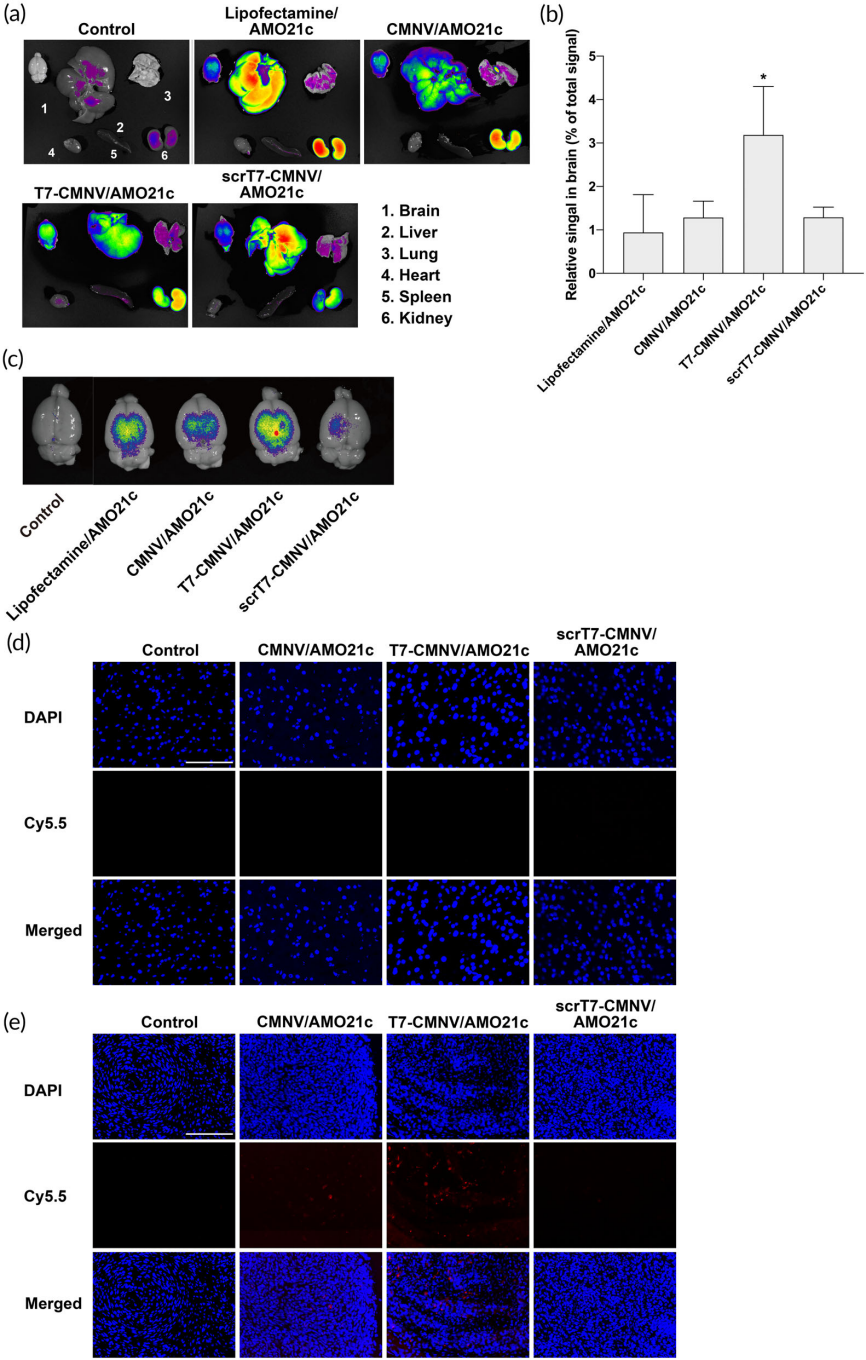
Delivery of AMO21c into the brains of animal models. Cy5.5‐AMO21c was delivered into rat glioblastoma models using lipofectamine, CMNV, T7‐CMNV, and scrT7‐CMNV through intravenous administration. The brain and other organs were harvested at 18 h after injection. Fluorescence images and quantitative analyses were obtained using an imaging box. (a) Representative fluorescence images of organs. Control groups were injected with PBS for blank subtraction. (b) Cy5.5‐AMO21c in the brain. The percentages of fluorescence signals versus total signals are presented as the mean ± standard deviation of triplicate experiments. *p < 0.05 as compared to the other groups. (c) Representative images of the brains. The tumor tissues (d) and normal brain tissues (e) were cut into 5‐μm‐thick sections. The Cy5.5 signals were observed by fluorescence microscopy. The nuclei were counter‐stained with DAPI. The scale bars indicate 100 μm.

The delivery efficiency of T7‐CMNVs was evaluated by studying their biodistribution. Lipofectamine, CMNVs, and scrT7‐CMNVs were used as negative controls. Cy5.5‐AMO21c was loaded onto CMNVs, T7‐CMNVs, and scrT7‐CMNVs by hydrophobic interaction. Also, lipofectamine/Cy5.5‐AMO21c was prepared by charge interaction. At 18 h after intravenous injection of the sample into the tail vein, the organs were harvested, and fluorescence signals were observed in an image box. Most of the AMO21cs were observed in the liver and kidneys. In particular, lipofectamine/AMO21c was taken up by the liver more highly than CMNVs with AMO21c (Figure 7a). This indicates that positively charged lipofectamine/AMO21c particles were taken up by Kupffer cells more easily than CMNVs. In contrast, CMNVs with negative surface charge may reduce the clearance by Kupffer cells. The fluorescence signals of Cy5.5‐AMO21c in the brain were increased by T7‐CMNV, compared with CMNV, scrT7‐CMNV, and lipofectamine (Figure 7b,c). The results confirmed that T7 peptides facilitated transcytosis and delivery efficiency into the brain. To evaluate the targeting effect, the fluorescence signals of Cy5.5‐AMO21c were observed in the normal brain and tumor tissues by fluorescence microscope. The results showed that the T7‐CMNV group showed higher positive signals in the tumors than the other groups, suggesting that T7 increased delivery of AMO21c into the tumors (Figure 7d). However, the signals were negligible in the normal brain tissue (Figure 7e).

It was previously suggested that tumor cell membrane‐coated nanoparticles have homotypic targeting effects; thus, CMNVs might have tumor‐targeting effects related to homotypic targeting.^29^ However, this effect was not observed in this study, and the delivery efficiencies of CMNVs and scrT7‐CMNVs were similar to lipofectamine, which did not have a brain targeting moiety. This may be because CMNVs cannot enter the brain through the BBB efficiently, and brain‐targeting ligands, such as T7 peptides, may be useful to increase the delivery efficiency.

Body weight, AST, ALT, and BUN were measured to evaluate the possible toxicity of CMNVs and T7‐CMNVs in vivo. The results indicated that CMNVs and T7‐CMNVs did not cause noticeable toxicity in the animals (Figure S2).

### 3.4 Tumor targeting delivery and therapeutic effects of AMO21c using T7‐CMNVs

**FIGURE 8:**
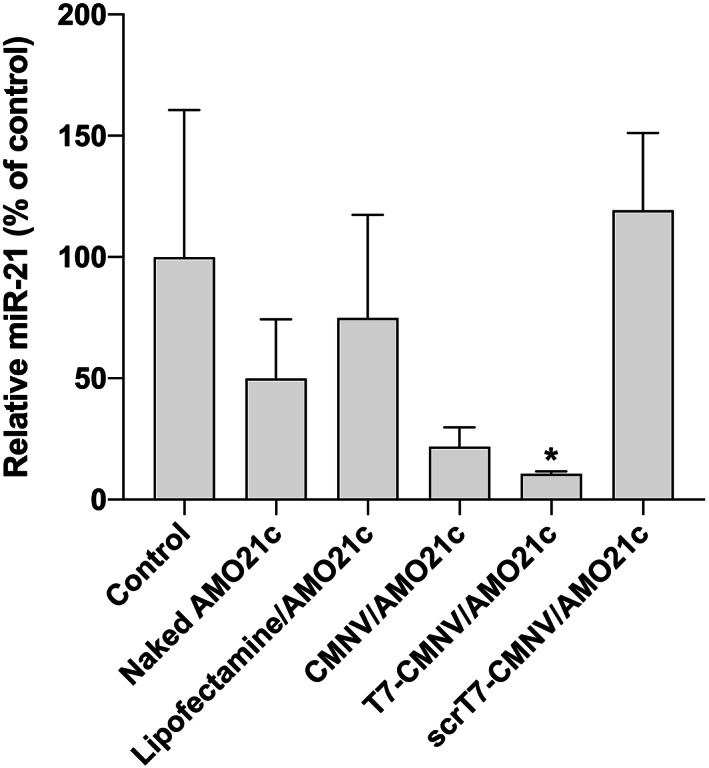
Suppression of miR‐21 by AMO21c delivery in animal models. Naked AMO21c, lipofectamine/AMO21c, CMNV/AMO21c, T7‐CMNV/AMO21c, and scrT7‐CMNV/AMO21c were prepared and injected into glioblastoma rat models via the tail vein. Downregulation of brain miR‐21 levels was measured with RT‐PCR. The data are expressed as the mean ± standard deviation of quadruplicate experiments. *p < 0.05 as compared to the control, lipofectamine/AMO21c, and scrT7‐CMNV/AMO21c groups.

Orthotopic glioblastoma models were produced by injecting C6 cells into the brains of rats. One week after implantation, T7‐CMNV/AMO21c was injected intravenously via the tail vein. Lipofectamine/AMO21c, CMNV/AMO21c, and scrT7‐CMNV/AMO21c were injected as controls. At 7 days after the injections, the animals were sacrificed, and the brains were harvested. To evaluate the effects of AMO21c delivery, we performed RT‐PCR to amplify miR‐21. The results showed that miR‐21 levels were decreased following the injection of CMNV/AMO21c and T7‐CMNV/AMO21c (Figure 8). In particular, the reduction of miR‐21 by T7‐CMNV/AMO21c was higher than that of CMNV/AMO21c. This result confirmed that T7‐CMNVs, compared to CMNVs, enhanced the delivery efficiency of AMO21c into the brain.

**FIGURE 9:**
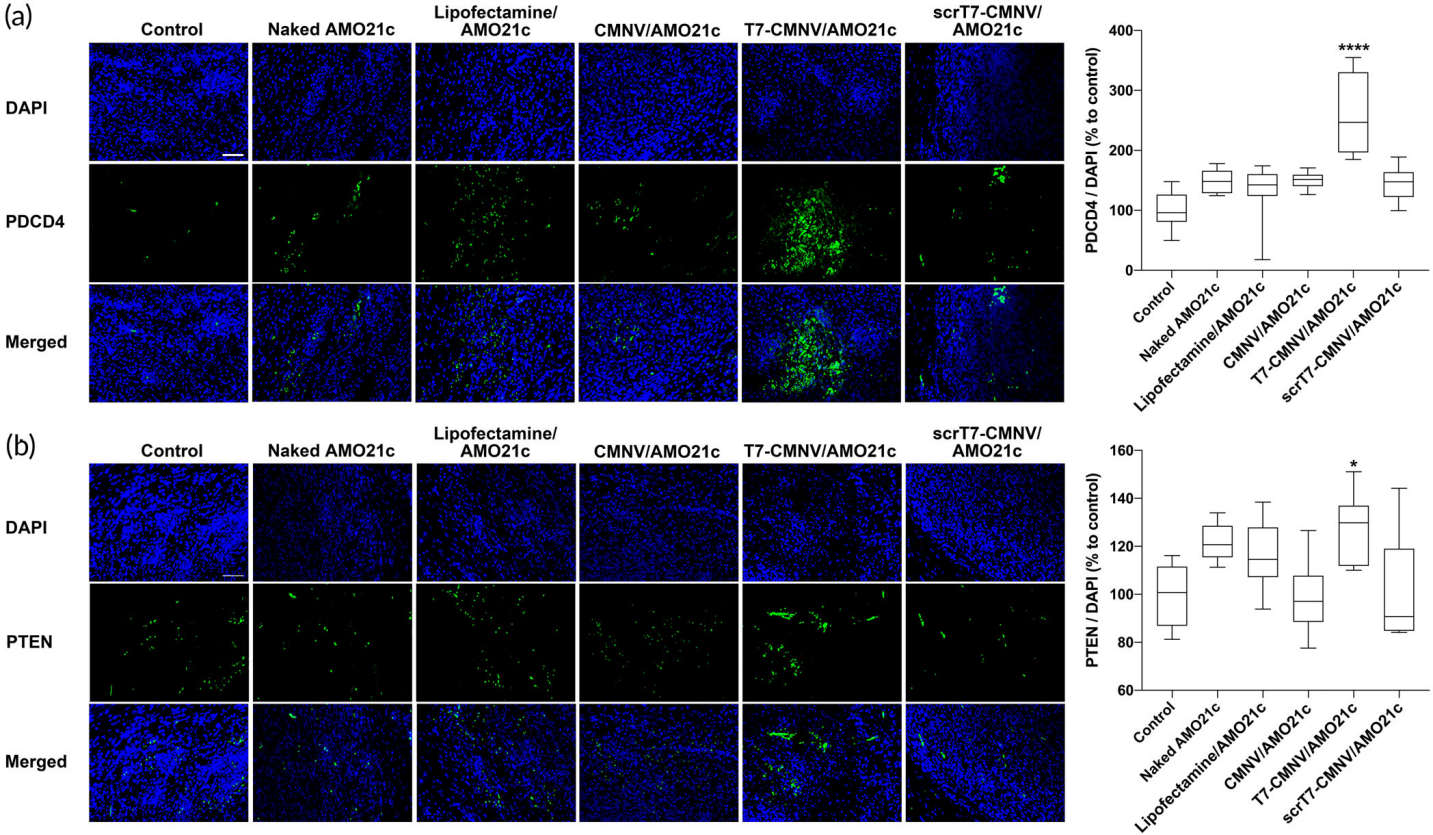
Induction of PDCD4 and PTEN by AMO21c delivery in animal models. Naked AMO21c, lipofectamine/AMO21c, CMNV/AMO21c, T7‐CMNV/AMO21c, and scrT7‐CMNV/AMO21c were prepared and injected into glioblastoma rat models via the tail vein. After 1 week, the brains were harvested and subjected to immunohistochemistry with (a) anti‐PDCD4 and (b) anti‐PTEN antibodies. Nuclei were counter‐stained with DAPI. Scale bars indicate 50 μm. The quantitation of mean fluorescence intensity was performed. The data are expressed as the mean ± standard deviation of decuplicate experiments. Relative expression levels were compared to the control group. ****p < 0.0001 as compared to the other groups. *p < 0.05 as compared to the CMNV/AMO21c and scrT7‐CMNV/AMO21c groups.

The reduction of miR‐21 may have an influence on its target gene expression. PDCD4 and PTEN expressions were evaluated in the brain samples to evaluate target gene expression. *PDCD4* and *PTEN* are target genes of miR‐21, which have miR‐21 recognition sites in their 3′‐UTRs. The down‐regulation of *PDCD4* and *PTEN* was closely related to the high survival rates of tumor cells in the tumor environment. Therefore, AMO21c delivery might inhibit miR‐21, resulting in an increase in PDCD4 and PTEN expression. The results showed that PDCD4 expression was increased by T7‐CMNV/AMO21c (Figure 9a). In particular, T7‐CMNV/AMO21c increased PDCD4 expression more efficiently than the other samples, including CMNV/AMO21c and scrT7‐CMNV/AMO21c (Figure 9a). As shown in Figure 6, CMNV/AMO21c did not penetrate the brain as efficiently as T7‐CMNV/AMO21c, suggesting that T7 peptides increased the delivery efficiency of AMO21c into the brain. Therefore, facilitated delivery of AMO21c by the T7 peptides of T7‐CMNVs may enhance PDCD4 expression in the brain. Similar results were observed in PTEN immunohistochemistry. PTEN expression was induced most efficiently by the intravenous administration of T7‐CMNV/AMO21c (Figure 9b).

**FIGURE 10:**
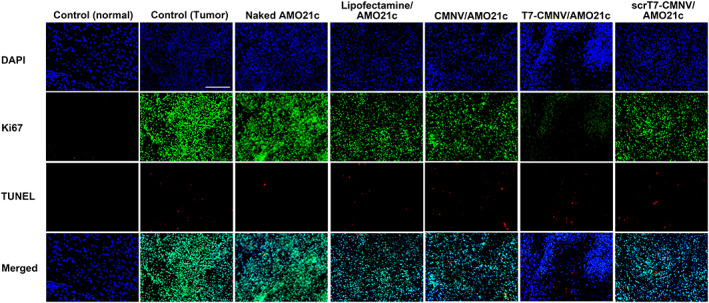
Growth inhibition and cell death. Naked AMO21c, lipofectamine/AMO21c, CMNV/AMO21c, T7‐CMNV/AMO21c, and scrT7‐CMNV/AMO21c were prepared and injected into glioblastoma rat models via the tail vein. After 1 week, the brains were harvested and subjected to immunohistochemistry with anti‐Ki67 antibody (green) and the TUNEL assay (red). Nuclei were counter‐stained with DAPI. The scale bar indicates 50 μm.

Several studies suggest that extent of proliferation marker Ki67 expression is strongly proportional to cancer severity.^30^, ^31^ To evaluate the anti‐tumor effects of AMO21c delivery, we evaluated Ki67 expression in the rat brain through immunohistochemistry. Lipofectamine/AMO21c, CMNV/AMO21c, and scrT7‐CMNV/AMO21c also reduced Ki67 expression (Figure 10). However, Ki67 expression was reduced more efficiently by T7‐CMNV/AMO21c (Figure 10). The enhanced reduction of Ki67 by T7‐CMNV/ AMO21c may be due to the more efficient delivery of AMO21c into the brain by T7‐CMNVs, supporting the evidence suggesting that T7 peptides on the surface of T7‐CMNVs facilitated the penetration of AMO21c into the brain through the BBB.

The expression of PDCD4 and PTEN might increase tumor apoptosis since the proteins are involved in growth arrest and apoptosis. To evaluate apoptosis levels, we performed the TUNEL assay with the brain samples. However, the apoptosis levels of the samples were not significantly different from one another (Figure 10). This unexpected result may be due to the characteristics of solid tumors. In the cores of glioblastoma, the cells are under hypoxic conditions. As a result, glioblastoma tumors have intratumoral necrosis and apoptosis at various levels.^32^, ^33^, ^34^ As the size of a solid tumor grows, the hypoxic region in the medial part of the cancer also expands, as it lacks nutrients and oxygen.^35^ Necrosis and apoptosis occur widely in the cores of both small and large tumors. Also, the rat glioblastoma models in this study had the apoptotic region in the core (data not shown). Thereby, it is likely that the apoptotic signals in the tumors could be observed not only by the anti‐cancer effects of AMO21c but also by hypoxia.

**FIGURE 11:**
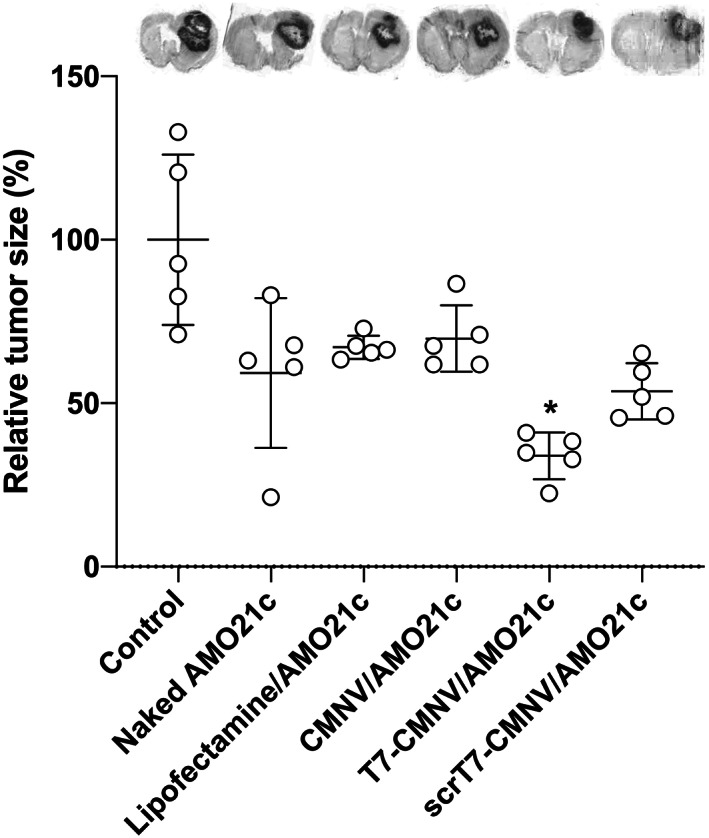
Tumor size. Naked AMO21c, lipofectamine/AMO21c, CMNV/AMO21c, T7‐CMNV/AMO21c, and scrT7‐CMNV/AMO21c were prepared and injected into glioblastoma rat models via the tail vein. After 1 week, the brains were harvested and subjected to Nissl staining. Relative tumor sizes are presented as % of the control group. The data are expressed as the mean ± standard deviation of quintuplicate experiments. *p < 0.05 as compared to the control, lipofectamine/AMO21c, and CMNV/AMO21c groups.

The tumor sizes of the samples were evaluated through Nissl staining. The tumor sizes across all groups treated with AMO21c were decreased. In particular, T7‐CMNV/AMO21c decreased the tumor size most efficiently compared with the other samples (Figure 11). Other groups also reduced tumor size by about 40%, but the effect was not as significant as T7‐CMNV/AMO21c. Specifically, CMNV/AMO21c and scrT7‐CMNV/AMO21c had lower effects on tumor size than T7‐CMNV/AMO21c, further suggesting the effect of T7 peptides on tumor targeting delivery through the BBB.

## 4 DISCUSSION

In this study, brain tumor‐targeting cell membrane‐based nanovesicles, T7‐CMNVs, were developed and evaluated as carriers of AMO21. In vitro and in vivo studies provided compelling evidence that T7‐CMNVs delivered AMO21c into brain tumors and suppressed tumor growth through miR‐21 inhibition. The results indicate that T7 peptides on T7‐CMNVs facilitated the penetration of CMNVs through the BBB.

Cell membranes have been intensively investigated within drug delivery systems in various forms. Cell membrane‐coated nanoparticles loaded with anti‐cancer drugs have been reported to exhibit tumor‐targeting effects. In particular, tumor cell membranes have been reported to have tumor‐targeting effects through homotypic targeting effects.^29^, ^36^, ^37^, ^38^ The membrane proteins on the tumor cell membrane interact with homotypic cell membranes, facilitating specific interactions. For example, doxorubicin‐loaded nanoparticles become coated with tumor cell membranes. Cell membrane‐coated nanoparticles have some advantages over noncoated nanoparticles. First, cell membranes reduce the interactions with serum proteins and the resulting opsonization.^39^ This may increase circulation time and bioavailability. Second, tumor cell membrane‐coated nanoparticles interact with homotypic tumor cells specifically, increasing delivery efficiency into the tumors. Third, the toxicity of nanoparticles may be reduced by coating them with cell membranes.^39^ Due to these advantages, cell membranes have been investigated for various purposes. Recently, nanovesicles with cell membranes were produced for drug delivery. Cell membrane‐based nanovesicles have exosome‐like properties. They were able to penetrate the BBB to deliver drugs into the brain. A recent report showed that doxorubicin, which is hydrophobic, can be loaded into the membranes of nanovesicles through hydrophobic interactions.^40^ The CMNVs delivered doxorubicin into brain tumors across the BBB.

Although many efforts were made to use CMNVs in a drug delivery system, the delivery of nucleic acid drugs, such as AMOs, has not been attempted with CMNVs. Delivery of AMO was previously attempted with exosomes. However, exosome‐mediated delivery of AMOs has some hurdles. The production of exosomes from mammalian cells is not efficient.^17^ Therefore, a large quantity of production for animal or human applications may be cost‐intensive. In addition, the contents of exosomes may cause deleterious effects. Therefore, the use of exosomes for clinical applications may be limited due to their side effects. Also, AMO loading into exosomes is not efficient. Physical methods such as electroporation or sonoporation have loading efficiencies as low as 1%–5%.^13^, ^25^ In contrast, CMNVs may be free from these problems. First, cell membranes can be isolated more easily than exosomes in a large quantity. For exosome purification, the cells should be cultured in exosome‐free medium to avoid any contamination from serum exosomes. Second, CMNVs from isolated cell membranes do not have undefined contents inside of CMNVs unlike exosomes. Third, membrane proteins on CMNVs originate from the source cells, which may contribute to homotypic targeting effects. Fourth, the AMOs can be easily loaded on the CMNVs by hydrophobic interactions. AMOs can be linked with hydrophobic cholesterol, which contributes to the integration of AMOs into the CMNVs. The loading efficiency was increased by this method up to 15%.

The loading of AMO21c onto T7‐CMNVs was conferred by the hydrophobic interaction between cholesterol and the cell membranes. The possible problems of cholesterol‐linked AMOs are cytotoxicity due to cholesterol, degradation of AMOs on the CMNV surface by enzymes, and interference with the targeting effects of T7 peptides. Some studies were performed to evaluate these possibilities. First, in vitro and in vivo toxicity assays show that AMO21c does not have any noticeable toxicity. This suggests that the cholesterol of AMO21c may not significantly induce toxicity. Second, the degradation of AMO21c was minimized by chemical modification of AMO21. Methyl groups were attached to the 2′‐hydroxyl group, which reduced nuclease‐mediated degradation. Third, AMO21c on the surface of CMNVs may interfere with the targeting effects of T7 peptides, but our in vivo results suggest that T7‐CMNVs still facilitated the delivery of AMO21c into glioblastoma tissue. However, the possibility that AMO21c on the surface of T7‐CMNVs may decrease the targeting effects of T7 peptides is not excluded.

Although CMNVs from tumor cell membranes were reported to have tumor‐targeting effects, the decoration of CMNVs with T7 peptides increased the delivery efficiency into the brain. This suggests that the decoration of targeting ligands on tumor cell membrane‐coated nanoparticles or other types of CMNVs may have similar effects on delivery efficiency. It was previously suggested that CMNVs might have exosome‐like properties, including penetration of the BBB. However, there is still room for improvement in delivery efficiency using specific targeting ligands.

The stability of T7‐CMNVs is important for translational applications. T7‐CMNVs may have high level of transferrin receptors on the surface, since they were produced with the cell membranes from C6 cells. T7 peptide on a T7‐CMNV may bind to the transferrin receptors on other T7‐CMNV. This inter‐CMNV interaction may induce aggregation by fusion of T7‐CMNVs. In Figure 2d, the stability of T7‐CMNVs was maintained for up to 21 days. However, it is possible that the size of T7‐CMNVs may be increased by incubation for longer than 21 days. To avoid this size increase, T7‐CMNVs may be prepared with cell membranes from the cells that have low transferrin receptors.

## 5 CONCLUSIONS

In summary, glioblastoma‐targeting CMNVs with targeting ligands were developed in the current study. The physical characterization of T7‐CMNVs suggests that T7‐CMNVs are quite stable in storage for more than 21 days. T7‐CMNVs delivered AMO21c into glioblastoma tissues across the BBB after intravenous administration. As a result, the enhanced delivery of AMO21c downregulated miR‐21 levels, leading to the restoration of genetic markers, such as PDCD4, PTEN, and Ki67. Overall cancer size was reduced significantly following treatment with T7‐CMNV/AMO21c. Taken together, these results suggest that T7‐CMNVs may be an efficient carrier for the delivery of AMO21c to treat glioblastoma.

## AUTHOR CONTRIBUTIONS

**Youngki Lee:** Data curation (equal); formal analysis (equal); investigation (equal); methodology (equal); writing – original draft (equal). **Minkyung Kim:** Investigation (equal). **Junkyu Ha:** Investigation (supporting). **Minhyung Lee:** Conceptualization (equal); data curation (equal); formal analysis (equal); funding acquisition (equal); methodology (equal); supervision (equal); writing – review and editing (equal).

## CONFLICT OF INTEREST

The authors declare no competing interest.

### PEER REVIEW

The peer review history for this article is available at https://publons.com/publon/10.1002/btm2.10426.

## Supporting information


**Figure S1.** Optimization of the ratio between CMNV and AMO21c. Various amounts of AMO21c were mixed with a fixed amount of CMNVs and incubated for 30 min at room temperature. CMNV/AMO21c were added to the cells, followed by incubation for 4 h. After changing the media, the cells were incubated in a 5% CO_2_ incubator for an additional 20 h. The cellular uptake of AMO21c was evaluated by flow cytometry. The delivery efficiency seemed to be saturated at a 1:5 weight ratio. Therefore, the ratio between CMNV and AMO21c was fixed at 1:5 for the following experiments. The data are expressed as the mean ± standard deviation of quadruplicate experiments. ****p < 0.0001 as compared to the control, naked AMO21c, 1:1.25, and 1:2.5 groups, but no statistical significance was observed for comparisons with 1:10 and 1:20 CMNV:AMO21c ratio groups.
**Figure S2.** Evaluation of in vivo toxicity. The toxicity of nanovesicles and lipofectamine evaluated by (a) body weight (*n* = 6), (b) aspartate aminotransferase (*n* = 3), (c) alanine transaminase (*n* = 3), and (d) blood urea nitrogen (*n* = 3).

## Data Availability

The authors confirm that the data supporting the findings of this study are available within the article and its supplementary materials or available from the corresponding authors upon reasonable request.
