# Supplementary material for: Brain‐targeted exosome‐mimetic cell membrane nanovesicles with therapeutic oligonucleotides elicit anti‐tumor effects in glioblastoma animal models
Source: Bioeng Transl Med. 2022 Oct 18;8(2):e10426. doi: 10.1002/btm2.10426 (PMC10013800; doi:10.1002/btm2.10426)
Supplement: Supplementary file 1 — Figure S1. Optimization of the ratio between CMNV and AMO21c. Various amounts of AMO21c were mixed with a fixed amount of CMNVs and incubated for 30 min at room temperature. CMNV/AMO21c were added to the cells, followed by incubation for 4 h. After changing the media, the cells were incubated in a 5% CO2 incubator for an additional 20 h. The cellular uptake of AMO21c was evaluated by flow cytometry. The delivery efficiency seemed to be saturated at a 1:5 weight ratio. Therefore, the ratio between CMNV and AMO21c was fixed at 1:5 for the following experiments. The data are expressed as the mean ± standard deviation of quadruplicate experiments. ****p < 0.0001 as compared to the control, naked AMO21c, 1:1.25, and 1:2.5 groups, but no statistical significance was observed for comparisons with 1:10 and 1:20 CMNV:AMO21c ratio groups. Figure S2. Evaluation of in vivo toxicity. The toxicity of nanovesicles and lipofectamine evaluated by (a) body weight (n = 6), (b) aspartate aminotransferase (n = 3), (c) alanine transaminase (n = 3), and (d) blood urea nitrogen (n = 3). [file BTM2-8-e10426-s001.docx]

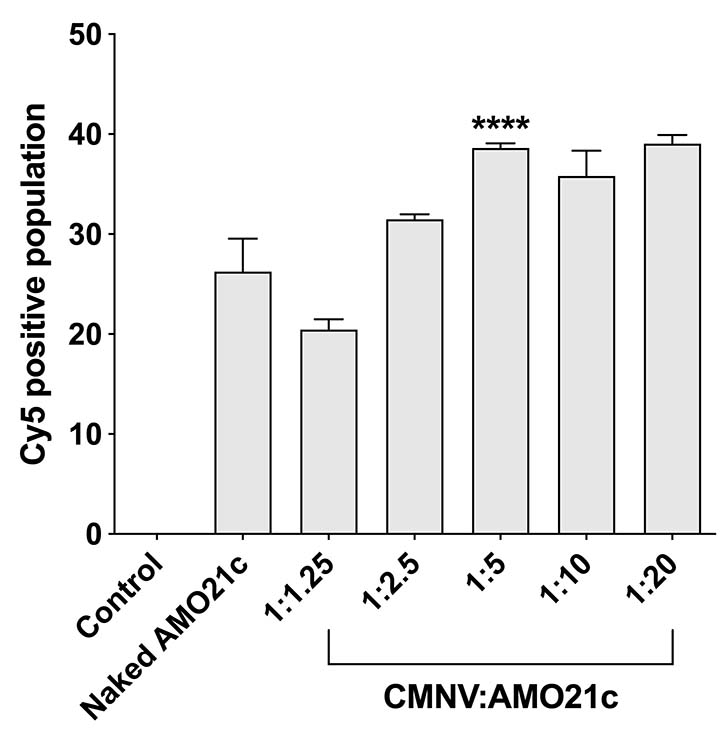


**Supplementary Fig. 1. Optimization of the ratio between CMNV and AMO21c.** Various amounts of AMO21c were mixed with a fixed amount of CMNVs and incubated for 30 min at room temperature. CMNV/AMO21c were added to the cells, followed by incubation for 4 h. After changing the media, the cells were incubated in a 5% CO_2_ incubator for an additional 20 h. The cellular uptake of AMO21c was evaluated by flow cytometry. The delivery efficiency seemed to be saturated at a 1:5 weight ratio. Therefore, the ratio between CMNV and AMO21c was fixed at 1:5 for the following experiments. The data are expressed as the mean ± standard deviation of quadruplicate experiments. ****P<0.0001 as compared to the control, naked AMO21c, 1:1.25, and 1:2.5 groups, but no statistical significance was observed for comparisons with 1:10 and 1:20 CMNV:AMO21c ratio groups.


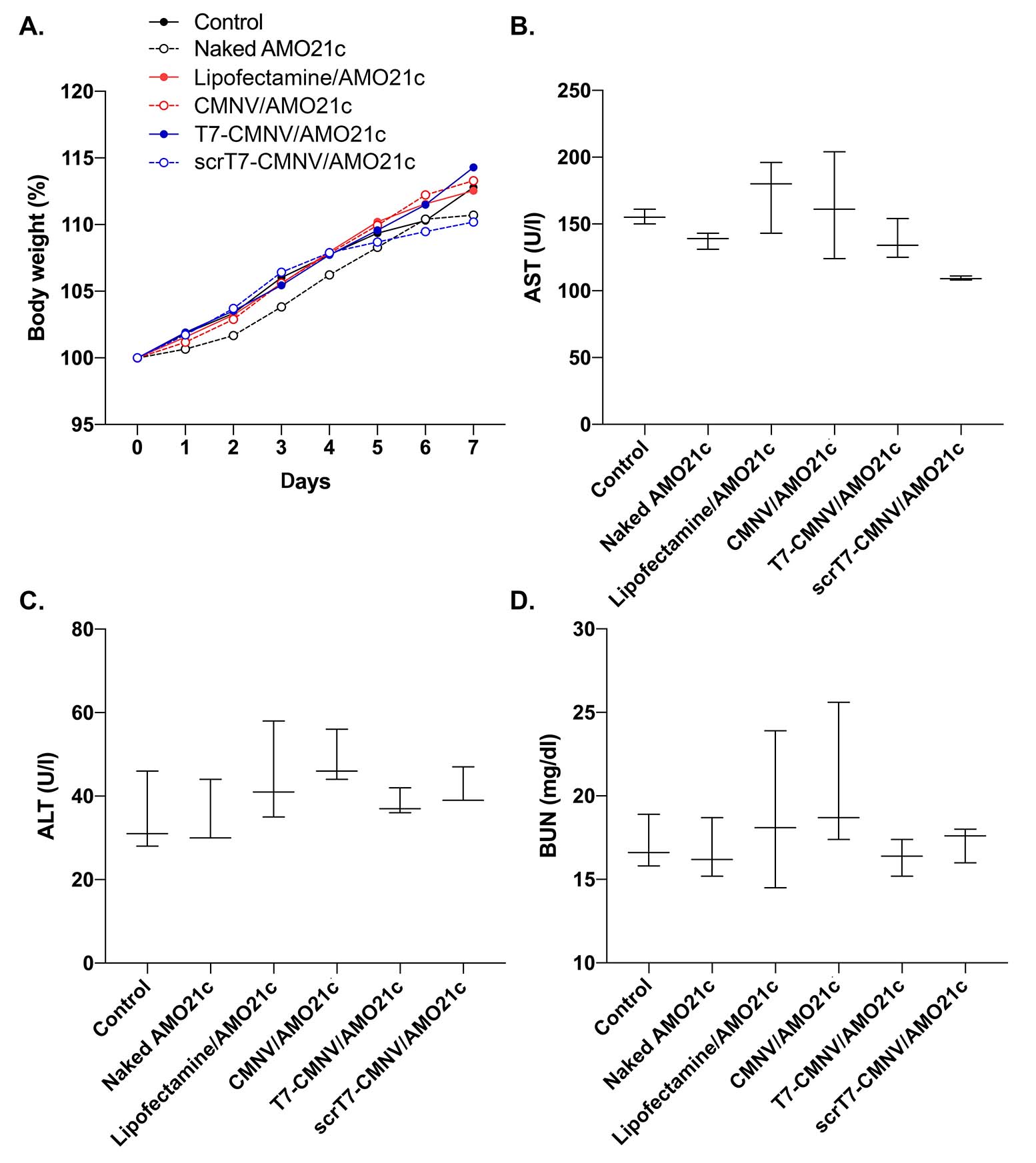


**Supplementary Fig. 2. Evaluation of *in vivo* toxicity.** The toxicity of nanovesicles and lipofectamine evaluated by **(A) body weight** (n=6), **(B) aspartate aminotransferase** (n=3), **(C) alanine transaminase** (n=3), and **(D) blood urea nitrogen** (n=3).
